# A Review on Ocular Novel Drug Delivery Systems of Antifungal Drugs: Functional Evaluation and Comparison of Conventional and Novel Dosage Forms

**DOI:** 10.34172/apb.2021.003

**Published:** 2020-11-07

**Authors:** Saba Mehrandish, Shahla Mirzaeei

**Affiliations:** ^1^Student Research Committee, Kermanshah University of Medical Sciences, Kermanshah, Iran.; ^2^Nano Drug Delivery Research Center, Health Technology Institute, Kermanshah University of Medical Sciences, Kermanshah, Iran.; ^3^Pharmaceutical Sciences Research Center, Health Institute, Kermanshah University of Medical Sciences, Kermanshah, Iran.

**Keywords:** Antifungal drugs, Conventional dosage forms, Novel drug delivery systems, Ocular drug delivery

## Abstract

Ocular fungal infections affect more than one million people annually worldwide. They can lead to impaired vision or even complete blindness, so they should be treated immediately to prevent such consequences. Although topical administration has always been the most common route of ocular drug delivery owing to high patient acceptance, reduced side effects, and the possibility of self-administration, its limited ocular bioavailability poses a major challenge. As a result, attention has recently been drawn to the design and development of novel drug delivery systems (NDDS) that can overcome the challenges of conventional dosage forms. This research is the first to review and classify the studies which have designed and developed topical ocular NDDS with the aim to compare the performance and antifungal activity of these novel systems with conventional forms. According to the results, all studies seemed to confirm the superiority of NDDS over conventional forms in cases of released and permeated drug and antifungal activity. The NDDS were used specifically to improve ocular delivery by slowing down the release rate, increasing drug permeation, and subsequently increasing the antifungal effects of the active pharmaceutical ingredients. Hence, further studies on NDDS may aid the optimization of ocular drug delivery of antifungal drugs.

## Introduction

The eye is a complex organ divided into two parts: the anterior and the posterior chambers, both of which can be affected by various diseases. Glaucoma, allergic conjunctivitis, uveitis, cataracts, and infections are the most common diseases affecting the anterior chamber.^1^ Ocular fungal infections have high prevalence, especially in developing countries with a hot and humid climate. According to the World Health Organization (WHO), corneal infections are one of the leading causes of vision loss.^2,3^ In recent decades, the prevalence of ocular fungal infections has increased due to the increased use of immunosuppressive agents such as steroidal drugs and broad-spectrum antibiotics as well as contact lenses and AIDS.^4,5^

Today, designing a system to deliver the drug at a therapeutic concentration to a specific tissue has become a major challenge for researchers. Different eye layers can create structural barriers against drug penetration. In addition, problems associated with drug properties, like water solubility, can challenge drug delivery. Sometimes, the only way to deliver the desired concentration of the drug to such an area is with injectable forms which are extremely invasive. Considering the benefits of topical forms, such as high patient compliance, ease of use, non-invasiveness, painlessness, reduced side effects, and selective treatment of the anterior chamber, topical ocular drug delivery has been one of the most effective and popular routes of administration from past to present day.^6-8^ Unfortunately, topical dosage forms face some challenges, like low drug penetration to the different layers of the eye, high frequency of administration, low residence time, and systemic toxicities caused by long-term use.^9,10^

Recently, many efforts have been made to improve topical ocular drug delivery by designing various novel drug delivery systems (NDDS), including liposomes, nanoparticles, nanoemulsions, nanosuspensions, micelles, nanofibers, etc.^10,11^ These systems can be used to optimize ocular drug delivery. In the present research, those studies which designed and prepared NDDSs to improve the ocular delivery of different antifungal drugs and compared them with conventional carriers in cases of released or permeated drug and antifungal activities were reviewed.

## Novel drug delivery systems

Synthesizing novel active pharmaceutical ingredients with a desirable efficacy has always been a challenge for scientists. Unfortunately, many recently designed compounds have been shown to be poorly soluble molecules with low permeability and high toxicity. As a result, scientists’ attention has been drawn to the design of NDDS.^12^ Generally, NDDS for ocular drug delivery are designed with two purposes: (1) make the drug controlled-release; and (2) increase its penetration to the cornea.^13^ These efforts have resulted in NDDS, like liposomes, nanoparticles, microemulsions, nanoemulsions, self-emulsifying systems, niosomes, nanosuspension, dendrimers, nanomicelles, nanofibers, hydrogels, etc.^10,12-14^

### Liposomes

Liposomes, initially introduced as bangosomes, are spherical, bilayer, closed systems formed by the suspension of phospholipids in water. These systems were first introduced in 1965 by Alec Bangham et al. Gregory Gregoriadis, a pioneer in the development of liposomal systems, was the first to consider liposomes as a suitable drug delivery system. Since that time, liposomes have become one of the most widely used NDDS. Today, a large number of commercial dosage forms have been formulated in liposomal systems. Liposomes can be prepared using cholesterol, sphingolipid, glycolipid, non-toxic surfactants, long-chain fatty acids, and membrane proteins. These systems can be used as carriers of small molecules, proteins, nucleotides, and drugs, and are classified based on size, structure, and charge.^15,16^ The main advantage of these systems is the possibility of encapsulating hydrophobic drugs in the phospholipid bilayer and hydrophilic drugs in the core of liposomes.^12,17^

In recent years, many studies have been done to evaluate and design liposomal systems for ocular drug delivery. One example is a 2010 study in which liposomal systems containing the antifungal drug fluconazole were prepared using reverse-phase evaporation and were formulated in drop form. To compare the conventional and liposomal form, both eyes of rabbits were inoculated with *Candida albicans* ; then, the right eyes of half of the rabbits were treated with the liposomal drop, while those of the other half were treated with fluconazole solution. The left eyes of the rabbits received no treatment and were considered as controls. The percentage of rabbits with completely cured right eyes increased by about 30% in the group treated with the liposomal drop compared to those treated with the conventional drug solution.^18^ In another study, the commercial 0.15% w/v drop of the antifungal drug Amphotericin B was compared with a liposomal form prepared by loading the drug in pre-prepared liposomes called AmBisome^®^. It was observed that the liposomal form was more stable and less toxic. The liposomal formulation also increased the potential amount of loaded drug by 3-fold compared with the conventional form.^19^ In a study performed by de Sá et al, a liposomal form was prepared by thin layer hydration technique using cholesterol, phosphatidylcholine, and 1,2-dioleoyl-3-trimethylammonium-propane to improve the ocular delivery of the antifungal drug voriconazole. The liposomal formulation, in addition to a sustained drug release profile, exhibited an 8-fold increase in the amount of drug retained in the cornea after 1 hour of exposure compared with the conventional suspension formulation.^20^ In 2015, itraconazole was loaded in a liposomal system prepared using cholesterol, soy phosphatidylcholine, and stearyl amine, by a thin-layer hydration method for the treatment of keratitis caused by *Aspergillus flavus*. Eyes of rats were infected with *Aspergillus flavus* and then divided into sample and control groups. The results were better in the group treated with the liposomal formulation than in the control group which was treated with pure drug. At the end of the treatment, no lesions in the eyes of 50% of the rats were seen in the group treated with the liposomal formulation, while in the group treated with the pure drug, no rat had completely cured eyes.^21^ A summary of these comparative results is shown in Table 1.

**Table 1:** Comparative studies conducted between conventional and liposomal formulations of antifungal drugs

**Drug name**	**Author and year of study**	**Summary of the results obtained**	**Reference**
Fluconazole	Habib et al, 2010	The percentage of rabbits with completely cured right eyes increased by about 30% in the group treated with the liposomal drop compared to those treated with the conventional drug solution.	^18^
Amphotericin B	Morand et al, 2007	The liposomal formulation also increased the potential amount of loaded drug by 3-fold compared with the conventional form, Fungizone^®^.	^19^
Voriconazole	De Sá et al, 2015	The liposomal formulation exhibited an 8-fold increase in the amount of drug retained in the cornea after 1 hour of exposure compared with the conventional suspension formulation.	^20^
Itraconazole	Leal et al, 2015	In the group treated with the liposomal form, 50% of the rats did not have any lesions in their eyes at the end of the treatment, while in the group treated with pure drug, there was no rat with completely treated eye.	^21^

### Nanoparticles

The introduction of nanotechnology was revolutionary in all industries. The importance and applicability of nanotechnology were first revealed in the early 1970s, following the introduction of a system that did not cause embolism after intravenous administration of a drug. Generally, nanoparticles are solid dispersed particles which can be polymers and of sub-micron size, preferably less than 500 nm. Based on the techniques used to prepare nanoparticles, they can be obtained in the form of nanospheres or nanocapsules. The first medical application of nanoparticles was in 1976 by Peter Speiser. The system was used to slow down antigen release for a better immune response in vaccination. Today, these systems are used for targeted and sustained drug delivery. Recently, multifunctional nanoparticles have been designed to deliver various drugs simultaneously in order to enhance therapeutic activity in addition to reducing side effects owing to the targeted delivery.^22^ Nanoparticles are of different types, such as magnetic, bioadhesive, gold, silver, solid lipid, self-aggregating, etc.^23,24^ These systems can also be formulated in film form to become suitable for prolonged ocular delivery.^25^

In a 2012 article, bio-adhesive nanoparticles containing the antifungal drug natamycin were prepared for ocular drug delivery using lecithin and chitosan by ionic gelation technique. The nanoparticle form, in addition to having a better pharmacokinetic profile, showed a two-step release pattern, i.e. an initial burst followed by a controlled release of drug in comparison to the conventional form of the drug. It was observed that the nanoparticle formulation released only 64% of the drug in 7 hours, while the pure drug released almost 100% of the drug in 2 hours. Also, the inhibition growth zone of the bioadhesive nanoparticle formulation against *C. Albicans* and *Aspergillus fumigates* was equal or greater than that of the conventional formulation.^26^ In another study, amphotericin B nanoparticles were prepared using polylactic acid-chitosan copolymer by dialysis. The nanoparticle formulation showed a sustained and controlled drug release for up to 11 hours, while the conventional drug formulation (0.15%) released all of the drug in only 4 hours. Nanoparticle formulation has also shown better pharmacokinetic properties, including a 1.95-fold increase in area under the curve and 1.5-fold increase in half-life compared to the conventional solution formulation.^27^ In 2013, Mohammed et al designed a nanoparticle formulation using chitin for ocular delivery of the antifungal drug fluconazole. The nanoparticle formulation showed a controlled, two-step release profile with only 15% of the drug released in the first 48 hours.^28^ Table 2 shows a summary of the results.

**Table 2:** Comparative studies conducted between conventional and nanoparticular formulations of antifungal drugs

**Drug name**	**Author and year of study**	**Summary of the results obtained**	**Reference**
Natamycin	Bhatta et al, 2012	Nanoparticle formulation had a controlled release profile and released only 64% of the drug in 7 hours, while the conventional form released almost 100% of the drug in 2 hours.	^26^
Amphotericin B.	Zhou et al, 2013	The nanoparticle formulation showed a sustained and controlled drug release for up to 11 hours, while the conventional drug formulation released all of the drug in only 4 hours.	^27^
Fluconazole	Mohammed et al, 2013	The nanoparticle formulation showed a controlled, two-step release profile with only 15% of the drug released in the first 48 hours	^28^

### Microemulsions, nanoemulsions, self-emulsifying systems

Emulsions are colloidal systems consisting of water and oil which have been thermodynamically stabilized by an emulsifier. Microemulsions, nanoemulsions, and self-emulsifying systems are three popular types of emulsions used for drug delivery. Nanoemulsions, first made in the 1940s, are emulsions with ultrafine globules ranging in size from 50 nm to 1000 nm; typically, however, emulsions with sizes below 200 nm are considered nanoemulsions.^29,30^ Microemulsions were made for the first time by Hoar and Schulman and were described as transparent, isotropic, and stable systems.^31,32^ Self-emulsifying drug delivery systems (SEDDS) are emulsions excluding the water phase which could prepare microemulsions ranging from 200 nm to 5 mm in size after exposure to water under agitation. Recently, attention has been drawn to SEDDS because of their ability to reduce the required dose of the drug in addition to their other benefits.^33^ Different types of emulsions have many advantages like the ability to be formulated into a variety of dosage forms, increase drug bioavailability, enhance stability, increase drug absorption due to a higher surface-to-volume ratio, and make lipophilic drugs more water-soluble. As a result, these systems are widely used in pharmaceutical and biological fields.^29^

In a 2017 study, a microemulsion formulation was prepared for ocular delivery of the antifungal drug fluconazole using isopropyl myristate as the oily phase and Tween 80 and polyethylene glycol 400 as surfactants. The microemulsion formulations showed a controlled release profile, releasing 50% to 80% of the drug in 12 hours, compared to the conventional drug solution which released almost all of the drug in the first 6 hours.^34^ In another study, a nanoemulsion formulation was designed for the ocular delivery of fluconazole using Capmul MCM as the oily phase and Tween 80 and Transcutol P as the surfactants with the spontaneous emulsification method. The nanoemulsion was then formulated to a gel form using Carbopol 934. The cumulative percentage of drug permeated to the goat’s cornea in the first 6 hours for the optimized nanoemulsion formulation was 3.71 times greater than that of the commercial drop formulation, Syscan^®^.^35^ In 2018, Elbahwy et al designed a thiolated self-emulsifying formulation by linking L-Cysteine, 6-mercaptonicotinamide, and Eudragit^®^ L100-55. The antifungal drug econazole was loaded in the designed system. The conventional solution of the drug released almost all of the drug in 4 hours, while the thiolated self-emulsifying formulation showed a two-step and controlled release profile, releasing only 60% of the drug in the first 8 hours.^36^ Table 3 shows a summary of these comparative studies.

**Table 3:** Comparative studies conducted between conventional and emulsion formulations of antifungal drugs

**Drug**	**Author and year of study**	**Summary of the results obtained**	**Reference**
Fluconazole	Soliman et al, 2017	The microemulsion formulations showed a controlled release profile, releasing 50% to 80% of the drug in 12 hours, compared to the conventional drug solution which released almost all of the drug in the first 6 hours.	^34^
Fluconazole	Pathak et al, 2013	The cumulative percentage of drug permeated to the goat's cornea in the first 6 hours for the optimized nanoemulsion formulation was 3.71 times greater than that of the commercial drop formulation, Syscan®.	^35^
Econazole	Elbahwy et al, 2018	The drug solution released almost all of the drug in 4 hours, while the self-emulsifying formulation released 60% of drug in the first 8 hours.	^36^

### Nanosuspensions

Nanosuspensions are colloidal dispersions of water-insoluble particles in water and have a particle size of less than 1 µm (according to some articles, less than 600 nm). These systems require a surfactant, polymer, or both to remain stable. The application of these systems in the field of pharmaceutics began in 1999, following the publication of an article by Muller et al. By 2005, five drugs with a nanosuspension formulation were approved by the FDA. Nanosuspensions are usually suitable for the delivery of poorly soluble drugs with good penetration. In addition to increasing the solubility and bioavailability of the drug due to the small particle size, these systems have the advantage of increasing mass-per-volume loading of the drug, which is useful, especially in cases where it is necessary to administer a high dose of drug or when the route of administration can provide the absorption of small amounts of drug (such as ocular and IM delivery). In addition to the prolonged and controlled release of drug, another advantage of these systems for ocular drug delivery is the increase in time the drug is retained in the cul-de-sac without increasing tonicity.^37-41^

In a 2016 study, nanosuspensions containing econazole were prepared using methyl-β-cyclodextrin and hydroxylpropyl-β-cyclodextrin and various stabilizers, such as polyethylene oxide, polyvinyl pyrrolidone k30, poloxamer 407, Tween 80, and Cremophor EL, with the nano-spray dryer technique. To prepare the nanosuspension formulation, nanoparticles were suspended in isotonic buffer and chitosan-hydrochloric acid solution. The *in vivo* study was performed on six Albino rabbits in each group using a parallel group design to compare the nanosuspension formulations with the suspension formulation. The nanosuspension formulations showed higher bioavailability and higher concentrations in the rabbit’s eye compared to the suspension formulation. In the suspension formulation, the concentration was measurable only up to 4 hours, while in nanosuspension formulations, the drug was still present in tear fluid up to 7 hours. Among the nanosuspension formulations, the chitosan-hydrochloric acid formulation with a concentration of 120 μg/mL in tears after 30 minutes showed a better performance compared to the buffer formulation with a concentration of 70 μg/mL.^42^ In another study, nanosuspension formulations were prepared using a mixture of itraconazole-chitosan with and without poloxamer. In the release study, it was observed that all nanosuspension formulations exhibited higher release compared to the conventional drug formulation. The optimized nanosuspension formulation exhibited a 2 times greater amount of released drug after 120 minutes compared to the suspension form.^43^ Table 4 shows a summary of these comparative studies.

**Table 4:** Comparative studies conducted between conventional and nanosuspension formulations of antifungal drugs

**Drug**	**Author and year of study**	**Summary of the results obtained**	**Reference**
Econazole	Maged et al, 2016	Nanosuspensions formulation exhibited a better performance with a 7-8 hours drug release compared to the suspension formulation with a 4 hours drug release.	^42^
Itraconazole	Ahuja et al, 2015	The optimized nanosuspension formulation exhibited a 2 times greater amount of released drug after 120 minutes compared to the suspension form.	^43^

### Niosomes

Niosomes are liposome-like systems with a higher chemical stability. The difference between these two systems is that, unlike liposomes which have a phospholipid structure, niosomes are bi-layer structures composed of nonionic surfactants.^44^ Niosomes can have particle sizes between 10 and 1000 nm, but more specifically, the term nanoniosome is used for particles below 100 nm in size.^45^ The main advantage of these systems can be their high flexibility in size and structure. Moreover, the surfactants used in these systems are mostly biodegradable, biocompatible, and non-immunogenic, which could make these systems suitable for drug delivery.^46^ Because of their amphiphilic nature, these systems have the potential to deliver lipophilic as well as hydrophilic drugs. So far, these systems have been used for the ocular delivery of timolol maleate, acetazolamide, and cyclopentolate.^44,45^

In a 2016 study, a niosomal system was prepared for the ocular delivery of fluconazole using Span 60, Span 80, and cholesterol with the film hydration technique. This system then was formulated to gel form using poloxamer and chitosan. In antifungal studies, the niosomal gel showed a 1.5- to 2-fold greater zone of inhibition compared to the conventional cream form. Moreover, in the drug release study, the niosomal gel formulation showed a controlled release profile by releasing only 30% to 50% of the drug after 6 hours compared to the solution form which had released 80% of the drug in the same time interval.^47^ In another study, a niosomal system was designed for ocular delivery of the antifungal drug voriconazole by film hydration method using Span 40, Span 60, Pluronic F127, Pluronic L64, and cholesterol. It was observed that the niosomal insert released the drug up to 8 hours, while the suspension form released all of the drug in 2 hours. The niosomal form also showed a 5.91-fold increase in bioavailability compared to the drug suspension, so that the amount of the drug in the aqueous humor after 1 hour of contact with the niosomal formulation was about 2 times greater than that of the drug suspension after 2 hours of contact.^48^ In 2019, Elnabarawi et al designed a niosomal formulation for ocular delivery of natamycin using cholesterol, Span 20, and dicetylphosphate by the reverse-phase evaporation technique. The niosomal form showed a controlled release profile, releasing only 20% to 50% of the drug in the first 10 hours compared to the commercial suspension formulation (NATACYN^®^5%) which released more than 90% of the drug in the same amount of time.^49^ Table 5 provides a summary of these studies.

**Table 5:** Comparative studies conducted between conventional and niosomal formulations of antifungal drugs.

**Drug**	**Author and year of study**	**Summary of the results obtained**	**Reference**
Fluconazole	Feithg et al, 2016	The niosomal gel formulation showed a controlled release profile by releasing only 30% to 50% of the drug after 6 hours compared to the solution form which had released 80% of the drug in the same time interval.	^47^
Voriconazole	Shukur, 2016	The niosomal insert released the drug up to 8 hours, while the suspension form released all of the drug in 2 hours.	^48^
Natamycin	Elnabarawi et al, 2019	The niosomal form showed a controlled release profile which released only 20% to 50% of the drug in the first 10 hours compared to commercial suspension formulation (NATACYN^®^ 5%) which released more than 90% of the drug in the same time.	^49^

### Micelles

Micelles are NDDS formed by amphiphilic molecules that can self-assemble in aqueous environments. The same molecules can create reversible micelles when placed in a non-aqueous medium that has an opposite orientation to typical micelles. The micelles can be formed in different shapes including spherical, cylindrical, or star-shaped, and can also be formed in sizes ranging from 10 nm to 1000 nm. Any amphiphilic molecule can form a micelle, but the concentration of surfactants should meet a specific standard, which is called the critical micelle concentration (CMC). Concentrations above CMC can lead to the formation of micelles, while the dilution of micelles in the blood and decreased surfactant concentrations can lead to the dissociation of micelles.^50,51^ Micelles with lower CMC exhibit higher thermodynamic stability.^52^ Many studies have been conducted to design these systems, especially nanosized micelles, as carrier and drug delivery systems for preventing drug destruction in the body and for targeting. Micelles can be used as drug delivery systems to improve the delivery of different drugs with different routes of administration, but they have been most widely used as carriers of anticancer drugs in various types, including multi-functional micelles, nanomicelles, and stimulus-responsive targeted nanomicelles.^52-54^

In a 2015 study, a micellar system containing itraconazole was developed using Pluronic F127 and F68 by the rotary evaporation method. In the penetration study, it was observed that the cumulative percentage of drug passed through the goat cornea from the optimized micellar formulation was 4-5 times greater than that of the commercial drop containing the drug suspensions (Itral^®^). In the antifungal study, the micellar formulation showed a zone of inhibition with a 4-cm greater diameter than that of the commercial suspension of the drug.^55^ In another study, a micellar system of terbinafine hydrochloride was made using macrogol 15 hydroxystearate with a simple co-solvent method. Pharmacokinetic studies showed that the corneal concentration of the drug in rabbit eyes was 3.9 times greater with the micellar formulation than with the oily solution.^56^ Younes et al developed a micellar system of the antifungal drug sertaconazole using Solutol HS 15, Pluronics, Brij 58, Transcutol P, and propylene glycol with the thin-film hydration method. Intracellular fluorescence analysis by confocal laser scanning showed that the optimized micellar formulation reached a 1.8 to 3.3 greater penetration depth to the rabbit cornea than the suspension formulation of the drug. Also, the micellar formulation showed a 340-times greater solubility compared to the suspension formulation, which could lead to improved penetration of the drug to ocular tissues.^57^ Table 6 shows a summary of these comparative studies.

**Table 6:** Comparative studies conducted between conventional and micellar formulations of antifungal drugs

**Drug**	**Author and year of study**	**Summary of the results obtained**	**Reference**
Itraconazole	Jaiswal et al, 2015	The optimized micellar formulation showed a 4-5 times greater cumulative percentage of the drug passed through the goat’s cornea compared to the commercial drop containing the drug suspensions (Itral^®^).	^55^
Terbinafine	Zhou et al, 2017	The corneal concentration of the drug in rabbit eyes was 3.9 times greater with the micellar formulation than with the oily solution.	^56^
Sertaconazole	Younes et al, 2018	The optimized micellar formulation reached a 1.8 to 3.3 greater penetration depth to the rabbit cornea than the suspension formulation of the drug.	^57^

### Nanofibers

Since the mid-1990s, attention has been drawn to these drug delivery systems. Researchers have already noticed the application of electrospun fibers, but in the 1990s, the importance of reducing the diameter of these fibers to nanoscales was noted.^58^ Although there are different methods for preparing nanofibers, including phase separation, drawing, and self-assembly, the electrospinning method has always been one of the most popular methods for preparing nanofibers. By applying a high voltage to an injected polymer solution, the droplets turn into a straight jet, which forms nanofibers when the solvent evaporates, and finally, the nanofibers are collected by a collector.^59^ Nanofibers are suitable systems for drug delivery. Their advantages include increased surface-to-volume ratio, high porosity, increased drug solubility, increased bioavailability of the drug, and specifically controlled drug release.^60,61^ Mirzaeei et al showed that nanofibers have the potential to release their drug in 4 days for ocular delivery, which could lead to less frequent administration and greater patient acceptance.^62^ Today, the application of these systems has increased to include healing agents.^63^

In a 2017 study, a nanofiber containing the antifungal drug voriconazole was designed using polyvinyl alcohol and hydroxypropyl-β-cyclodextrin by electrospinning. Pharmacokinetic studies showed improved kinetic with a 2.5-times greater bioavailability and 8-times greater half-life for the nanofibers compared to the solution formulation. Moreover, the nanofiber formulation showed a prolonged release in 24 hours, while the drug solution released all of the drug in 4 hours.^64^ Table 7 shows a summary of this comparative study.

**Table 7:** Comparative studies conducted between conventional and nanofiber formulations of antifungal drugs

**Drug**	**Author and year of study**	**Summary of the results obtained**	**Reference**
Voriconazole	Sun et al. 2016	The nanofiber formulation showed a prolonged release in 24 hours, while the drug solution released all of the drug in 4 hours.	^64^

### Hydrogels

The use of hydrogels has been considered since the 1960s following the study performed by Witchterle et al.^65^ These systems are net structures composed of water-soluble polymers which can have various chemical and physical properties. They have the ability to swell and absorb a high amount of water without being soluble.^66,67^ High porosity and the ability to load high levels of drug into these systems as well as the ability to absorb and release water have made these systems powerful for drug delivery. These systems can also control and prolong drug release. In many articles, these systems have been prepared to improve drug delivery. To date, these systems have been used for oral, anal, vaginal, ophthalmic, and transdermal drug delivery. Hydrogels are also used in tissue engineering, the manufacture of hygiene products, wound dressings, etc.^68^ Stimuli-responsive hydrogel with the ability to release the drug in response to a stimulant has also been designed to achieve more targeted drug delivery.^69^

A 2018 study designed a hydrogel system to improve ocular drug delivery of the antifungal drug econazole using cyclodextrins, hyaluronic acid, carrageenan, and gellan gum. Hydrogel formulations exhibited a controlled release profile by releasing the drug with a 1.5- to 3-times slower drug release rate in the first 24 hours compared to the solution formulation.^70^ In another study, bilosomal systems containing the antifungal drug natamycin were prepared using the film hydration method and a mixture of lipids. Then the hydrogels were obtained by adding gellan gum and xanthan gum.^71^ The *in situ* hydrogel formulations showed a 6-times greater drug permeation through the rabbit cornea compared to the commercial suspension of the drug (NATACYN^®^ 5%). Table 8 presents a summary of these comparative studies.

**Table 8:** Comparative studies conducted between conventional and hydrogel formulations of antifungal drugs

**Drug**	**Author and year of study**	**Summary of the results obtained**	**Reference**
Econazole	Díaz-Tomé et al, 2018	Hydrogel formulations exhibited a controlled release profile by releasing the drug with a 1.5- to 3-times slower drug release rate in the first 24 hours compared to the solution formulation.	^70^
Natamycin	Janga et al, 2018	The in situ hydrogel formulations showed a 6-times greater drug permeation through the rabbit cornea compared to the commercial suspension of the drug (NATACYN® 5%).	^71^

## Classification of the reviewed articles

The performance of novel and conventional drug delivery systems has been compared using one or more of the following methods in the reviewed studies:

*First group:* Amount of drug released, drug permeation to the cornea, retained drug in the cornea, depth of permeation in the eye.

*Second group:* The potential of the system to treat fungal infections *in vivo* or evaluation of antifungal activity *in vitro* by measuring the diameter of the inhibition growth zone.

*Third group:* Release profile and rate of drug release.

**Figure 1:**
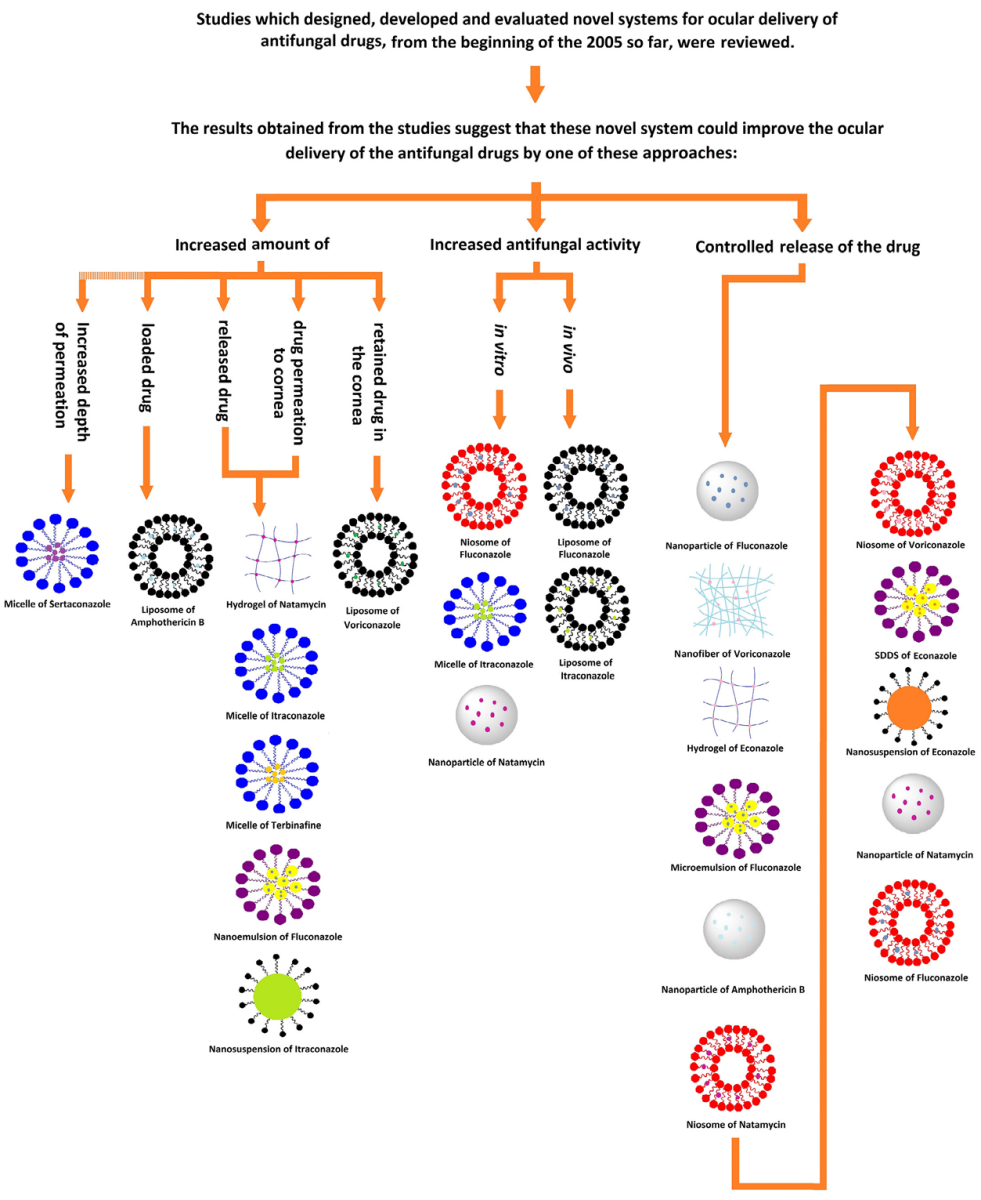
Schematic summary of the review.

In all of the reviewed studies with each of these comparative methods, NDDS were superior to conventional drug delivery systems in both released or permeated drug and antifungal activities. In the first group, which in most cases showed the percentage of penetrated or released drug, the highest increases were observed for a hydrogel (6-fold increase in drug permeation), a micelle (4-5-fold increase in cumulative percentage of the drug passed through the goat cornea), and a nanoemulsion (3.71-fold increase in cumulative percentage of drug permeated to the goat cornea). In the second group, the diameter of the growth zone was observed in most cases, with the largest increase in the niosome system (1.3-cm increase in diameter of zone of inhibition). There were few comparative studies in this category. In the third group, the longest release was observed with nanoparticles (48 hours of measured drug release), niosomes (24 hours of measured drug release), and nanofibers (24 hours of measured drug release) (Table 9). Still, it should be noted that in the third category, the drug delivery systems could not be ranked properly, because in most cases, the drug release was observed only for a certain time, and the study ended without stopping the drug release. In fact, the drug delivery systems probably released the drug for a longer time interval than mentioned (Figure 1).

**Table 9:** Classification of the studies

**Group of classification**	**Factor**	**Drug**	**System**	**Performance of novel system**	**Reference**
First group	Permeation and release through the cornea	Natamycin	Hydrogel	6 fold increase in drug permeation compared to the commercial suspension.	^71^
		Itraconazole	Micelle	4-5 fold increase in cumulative percentage of the drug passed through the goat’s cornea compared to the drug suspension.	^55^
		Terbinafine	Micelle	3.9 fold increase in corneal concentration of the drug in the rabbit's eye compared to the oily solution.	^56^
		Fluconazole	Nanoemulsion	3.71 fold increase in cumulative percentage of drug permitted to the goat's cornea compared to the commercial drop Syscan^®^.	^35^
		Itraconazole	Nanosuspension	2 fold increase in amount of released drug compared to the drug suspension.	^43^
	Depth of permeation in the eye	Sertaconazole	Micelle	1.8-3.3 fold increase in penetration depth of drug to the rabbit's cornea compared to the suspension formulation.	^57^
	Loading potential	Amphotericin B	Liposome	3 fold increase in loading potential compared to the commercial drug solution, Fungizone^®^.	^19^
	Retained drug in the cornea	Voriconazole	Liposome	8 fold increase in the amount of drug retained in the cornea compared to the conventional formulation.	^20^
Secondgroup	Treated ocular fungal infection	Fluconazole	Liposome	30 increase in percentage of completely treated rabbits compared to the conventional solution.	^18^
		Itraconazole	Liposome	50 increase in percentage of rats with completely treated eye compared to the conventional solution.	^21^
	Diameter of inhibition growth zone	Fluconazole	Niosome	1.5-2 fold decreased MIC for the novel formulation compared to the conventional form.	^47^
		Itraconazole	Micelle	0.4 cm increase in diameter of zone of inhibition compared to the conventional form.	^55^
		Natamycin	Nanoparticle	0.1 cm increase in diameter of zone of inhibition compared to the conventional form.	^26^
Third group	Release rate and profile	Fluconazole	Nanoparticle	Prolonged release with an almost 15% release in 48 hours for the novel formulation.	^28^
		Natamycin	Niosome	Prolonged release with an almost 30-70% release in 24 hours for the novel formulations.	^49^
		Voriconazole	Nanofiber	Prolonged release with an almost 100% release in 24 hours for the novel formulation.	^64^
		Econazole	Hydrogel	Prolonged release with an almost 200-400 µg/cm^2^ released drug in 24 hours for the novel formulations.	^70^
		Fluconazole	Microemulsion	Prolonged release with an almost 60-80% release in 12 hours for the novel formulations.	^34^
		Amphotericin B	Nanoparticle	Prolonged release with an almost 70-80% release in 11 hours for the novel formulations.	^27^
		Voriconazole	Niosome	Prolonged release with an almost 40-60% release in 8 hours for the novel formulations.	^48^
		Econazole	SDDS	Prolonged release with an almost 50-80% release in 8 hours for the novel formulations.	^36^
		Econazole	Nanosuspension	Prolonged release with high concentration in tear for 7-8 hours in the novel formulation.	^42^
		Natamycin	Nanoparticle	Prolonged release with an almost 60% release in 7 hours for the novel formulation.	^26^
		Fluconazole	Niosome	Prolonged release with an almost 30-50% release in 6 hours for the novel formulation.	^47^

There are a few FDA-approved NDDSs like Amphotec, Ambisome™, Abelcet™, Amphocil™, and Fungizone which are liposomal injectable formulations of amphotericin B for the treatment of invasive fungal infections. Nyotran^®^ is also a lyophilized liposomal injectable formulation containing the antifungal drug nystatin.^12^ To date, however, there is no FDA-approved novel topical ocular antifungal delivery system available.

## Conclusion

As previously mentioned, the prevalence of ocular fungal infections has increased recently; however, conventional topical drug delivery systems usually do not show a suitable performance in the treatment of these fungal infections due to challenges related to ocular drug delivery. As a result, these drugs require a long duration of treatment with a high frequency of drug administration, which is neither safe nor easily accepted by the patient. Researchers’ attention has recently been drawn to the development of NDDS. This study was the first to review and classify those studies which have designed and developed topical ocular NDDS aimed at comparing the performance and antifungal activity of these novel systems with conventional forms. Based on the results, it was found that all of the NDDS were superior to conventional systems in cases of released and permeated drug and antifungal activity. These systems had the ability to make the drug sustained-release, increase drug permeation and release, and subsequently increase the antifungal effects of the drug. It should be noted, however, that each of these systems can improve drug delivery with a specific approach. For example, nanofibers usually have the ability to prolong drug release; nanoemulsions usually have the potential to increase drug penetration to the target site. Many of these systems can improve drug delivery with more than one approaches; however, due to the variation of results in different comparative studies, a specific drug delivery system cannot be considered as the best. Finally, given the proven benefits of NDDS, further studies are needed to optimize these systems for commercial production and make them available for patient.

## Ethical Issues

Not applicable.

## Conflict of Interest

There is no conflict of interest.

## Acknowledgments

The authors would like to acknowledge the Research Council of Kermanshah University of Medical Sciences (Grant number: 96671) for financial support of this work.
